# Splitting CO_2_ in Intense Pulsed Plasma Jets

**DOI:** 10.3390/ijms24086899

**Published:** 2023-04-07

**Authors:** Adrian Scurtu, Dorina Ticoş, Maria Luiza Mitu, Constantin Diplașu, Nicoleta Udrea, Cătălin Mihai Ticoș

**Affiliations:** 1 National Institute for Laser, Plasma and Radiation Physics, Atomistilor Street 409, 077125 Măgurele, Romania; 2 Horia Hulubei National Institute for R&D in Physics and Nuclear Engineering, 077125 Măgurele, Romania

**Keywords:** CO_2_ splitting, dissociation, plasma jets, coaxial plasma gun

## Abstract

The splitting of CO_2_ was studied in a pulsed plasma discharge produced in a coaxial gun at voltages between ~1 and 2 kV and peak discharge currents of 7 to 14 kA. The plasma was ejected from the gun at a speed of a few km/s and had electron temperatures between 11 and 14 eV with peak electron densities ~2.4 × 10^21^ particles m^−3^. Spectroscopic measurements were carried out in the plasma plume produced at pressures between 1 and 5 Torr, and evidence of CO_2_ dissociation into oxygen and CO was found. An increased discharge current led to the observation of more intense spectra lines and the presence of new oxygen lines, which implies more dissociation channels. Several dissociation mechanisms are discussed, the main candidate being the splitting of the molecule by direct electron impact. Estimates of dissociation rates are made based on measured plasma parameters and interaction cross-sections available in the literature. A possible application of this technique is in future Mars missions where the coaxial plasma gun running in the atmosphere could be able to produce oxygen at a rate of the order of over 100 g per hour in a highly repetitive regime.

## 1. Introduction

The dissociation of carbon dioxide is gaining interest among academic, industrial, and government communities as its presence as a waste product increases global warming [1,2,3]. Conversion of CO_2_ into value-added [4] chemicals or new fuels [5] is a priority for a scientist to minimize its negative effects. In recent years, CO_2_ recycling engineering has grown in interest, and various technologies have emerged, including thermolysis and thermochemical cycles [6], electrolysis [7], and photo-electrolysis or bio-fixation [8]. Compared to these techniques, the use of non-thermal plasmas could be advantageous in terms of efficiency [9] compared to thermal dissociation. In addition, recent research on CO_2_ recycling suggests integration into an electrical grid, although at present, from an economic point of view, it lacks competitiveness [10,11]. To mitigate the high level of CO_2_, scientists are turning to numerous new ideas, including the sequestration of CO_2_ into the oceans [12].

Some technological proposals to dissociate CO_2_ that compete for better efficiency are found in peer review and patent literature [13,14]. We mention here the thermal dissociation reactors with zirconia membrane and argon, which has a small percentage (0.5%) of dissociation [15,16], carbon nano-materials [17], and dielectric barrier discharge plasma which works only around supercritical CO_2_ states [18,19,20]. Other approaches, such as the Boudouard reaction, have been discarded as they require very high temperatures [21], and the CO_2_ solid phase requires special temperature and pressure conditions [5,22,23]. Carbon dioxide was also split by high-energy laser beams [24,25,26,27,28], and dissociation at high temperatures [29,30,31,32,33] in a plasma torch [34] generated a plasma with a fairly low density (10^13^ particles cm^−3^). Alternative solutions from the field of plasmas include gliding arc discharge reactors [35,36], glow discharges [37], microwave discharges [38,39], and inductively-coupled radiofrequency plasma [40].

One space application is the production of oxygen on Mars for future missions with humans. In order to be viable and sustain such large missions, the methods for converting CO_2_ into oxygen must be reliable and efficient from the point of view of energy consumed. The Martian atmosphere is made of CO_2_ (95.9%), Ar (1.9%), N2 (1.9%), and traces of other gases. Carbon dioxide can be converted into O_2_ for life necessities, and CO can be used as a propellant [41,42,43] for a space vehicle.

As an example, MOXIE is a device that successfully demonstrated the production of oxygen directly from the Martian atmosphere at a rate of 10 g per hour, similar to a small tree on Earth [44,45]. MOXIE works by compressing the gas intake and heating it to a high temperature (~800 °C) and then breaking down electrochemically the CO_2_ into oxygen and CO.

We propose a new dissociation technique of CO_2_ based on the use of a pulsed coaxial plasma gun. Basically, two tungsten electrodes, a long, centered rod, and a coaxial outer cylindrical shell are powered at voltages between 0.8 to 2 kV. The gun electrodes are installed inside a vacuum chamber and immersed in CO_2_ at a pre-set pressure, in our case, between 1 and 5 Torr. The gas is ionized into a plasma with electron temperatures up to 14 eV, well above the CO_2_ dissociation threshold, and can split the molecules into their components. An analysis of the emission spectra shows evidence of multiple species formed into the discharge.

The plasma produced In our coaxial gun accelerator is unique in terms of its parameters and is one of the most energetic electrical discharges when compared to other plasma types: it features a high electron density (~10^21^ particles m^−3^), high electron temperatures > 10 eV, and significantly long pulses of a few hundreds of microseconds.

Originally designed to obtain nuclear fusion by achieving high-density plasmas [46], the coaxial gun with cylindrical geometry has rapidly become a fundamental research tool in plasma physics to investigate physical properties such as magneto-hydrodynamic instabilities, flux ropes, or magnetic reconnection phenomena [47]. It can also be utilized successfully in technological applications. The acceleration of microparticles at high speeds [48,49], the fuel loading of tokamaks [50], and dense plasma injection of targets in nuclear fusion experiments [51,52,53,54,55] are examples of its use. In space applications, recent studies show the possibility of using plasma jets in a Mars-like environment [56,57]. On Mars, exploration probes suffer from dust storms that can cover their solar cells with dust. Pulsed plasma jets produced directly into a CO_2_ atmosphere could potentially be used for cleaning such dusty surfaces [57,58]. Additionally, in the field of space propulsion applications, some designs adopted the coaxial plasma gun [59,60].

## 2. Results

The predominant elements associated with the observed spectral lines are O+, O, CO_2_, O++, and CO, but we also detected some lines of W, which are found in the coaxial electrodes.

**Figure 1:**
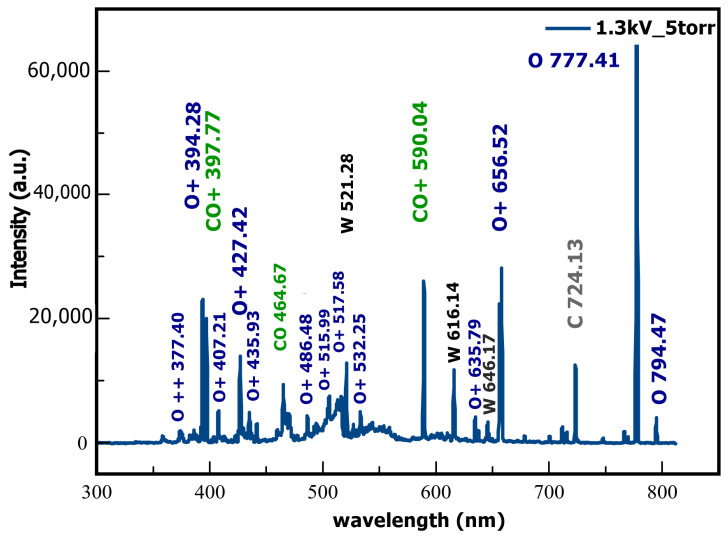
Emission spectra acquired during a shot at a discharge voltage of 1.3 kV and gas pressure inside the coaxial plasma gun of 5 Torr.

In Figure 1, we can see the most intense lines measured by the spectrometer when the coaxial plasma gun is operated at 1.3 kV and a pressure of 5 Torr inside the enclosure. One can recognize oxygen ions and atomic lines, such as O+ (394.28 nm), O+ (427.42 nm), O+ (656.52 nm), and O (777.41 nm), but also carbon monoxide CO (397.77 nm), CO+ (590.04 nm), and C+ (724.13 nm). The observed tungsten signatures are the lines W (521.28 nm) and W (616.14 nm).

**Figure 2:**
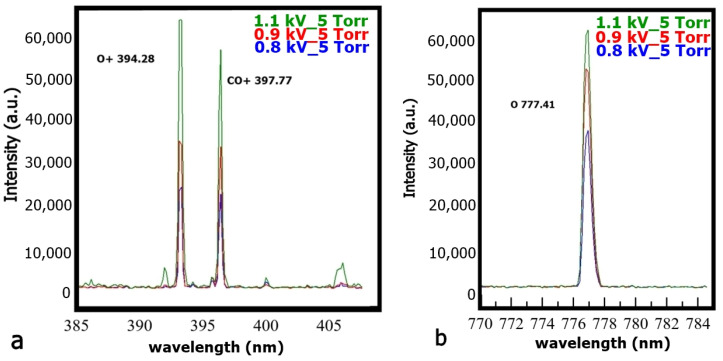
Evolution of the relative intensity of some oxygen and CO lines at different discharge voltages: (**a**) O+ (394.28 nm) and CO+ (397.77 nm) for 0.8 kV, 0.9 kV, and 1.1 kV, respectively, at a pressure of 5 Torr. (**b**): O (777.41 nm) line for 0.8 kV, 0.9 kV, and 1.1 kV, respectively, at the same CO_2_ pressure of 5 Torr.

**Figure 3:**
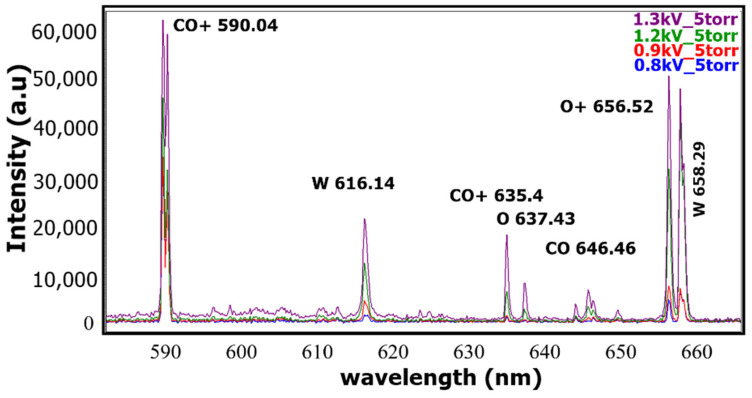
Evolution of the relative intensity of some oxygen, CO, and W lines when the discharge voltage is increased from 0.8 kV to 1.3 kV at the same CO_2_ pressure of 5 Torr.

An interesting observation is that the relative intensity of some spectral lines, such as those of O+ (394.28 nm) and CO+ (397.77 nm), increases by almost a factor of 3, whereas that of atomic oxygen (O 777.41 nm) shows a more moderate increase, with a factor of 1.5 when the discharge voltage is raised by ~37% from 0.8 kV to 0.9 kV and finally, 1.1 kV, as shown in Figure 2a,b. The same trend is seen in Figure 3, where the line intensity of the W (658.29 nm) and O^+^ line (656.52 nm) increases fivefold; the other CO+ lines (590.04 nm and 635.40 nm), the neutral oxygen (637.43 nm), and the W line (616.14 nm) increase by a factor of 3 while the CO line (646.46 nm) increases moderately by a few tens of percents.

**Figure 4:**
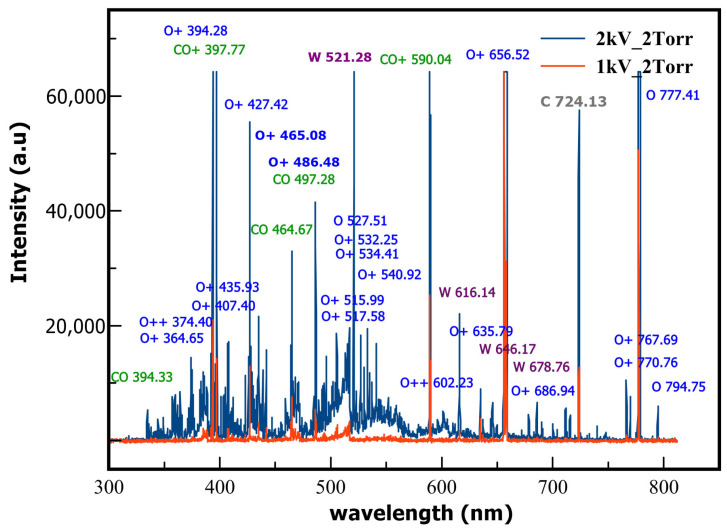
Full emission spectra captured for two discharge voltages, 1 kV (in red) and 2 kV (in blue), at a CO_2_ pressure of 2 Torr.

We also acquired the full emission spectra at 1 kV and 2 kV but at a slightly lower CO_2_ pressure of 2 Torr, as shown in Figure 4. One can see not only an increase in the relative intensity of the ion lines, such as O+ (394.28, 465.08, and 427.42 nm), CO+ (397.77 and 590.04 nm), and of the neutral O (777.41 nm), but also we detected new lines at the higher voltage. Such new lines belong to the neutral oxygen O (794.75 nm) single ionized oxygen O+ (364.65, 407.21, 435.93, 532.25, 534.41, 676.94, 767.69, and 770.67 nm) and doubly ionized oxygen O++ (374.400, and 602.232 nm). This is probably a result of multiple CO_2_ dissociation channels, which are induced by the higher electron density in the plasma.

In fact, we found that at a CO_2_ pressure of 5 Torr, the electron density increases from a peak value of $n_{e}^{1kV}=\left(1.7\pm 0.2\right)\times 10^{21}$ particles m^−3^ to $n_{e}^{2kV}=\left(2.4\pm 0.2\right)\times 10^{21}$ particles m^−3^ (as shown later in the Figures 10 and 11 of the Section 3.2). The discharges are characterized by two operating stages in time: the first stage has a duration of ~100 µs during which a hot electron population is produced, while in the second stage, during the time period of 150–200 µs, the electron population has a lower temperature. Thus, for the 1 kV discharge, the peak electron temperature reaches $T_{e}=11\pm 1$ eV in the first stage and drops to $T_{e}=3–5$ eV in the second stage.

In the case of a discharge at 2 kV, the peak electron temperature is $T_{e}=14\pm 1$ eV in the first stage, while in the second stage, the population of electrons cools down to about $T_{e}=7–9\;$eV. Apparently, the peak electron density drops by a factor of 2 when the discharge evolves between these two stages. Given the lower temperature of the secondary population of electrons, one can assume that it triggers different dissociation mechanisms.

The presented spectra are associated with the composition of the gas inside the chamber and also of the coaxial gun material, excluding contributions from impurities or other sources. The experimental enclosure is vacuumed down to a base pressure of ~10^−5^ Torr before high-purity CO_2_ is injected. Nevertheless, there is a possibility to find traces of nitrogen and hydrogen from water vapors that are desorbed from the electrodes but at levels that are well below the main peaks seen in our spectra. The high pulsed currents passing through the discharge circuit produce Joule heating and raise the temperature of the electrodes by tens of degrees. We carried the measurements at a CO_2_ pressure relevant to Mars’ atmosphere.

## 3. Discussion

### 3.1. Mechanism of Dissociation

**Figure 5:**
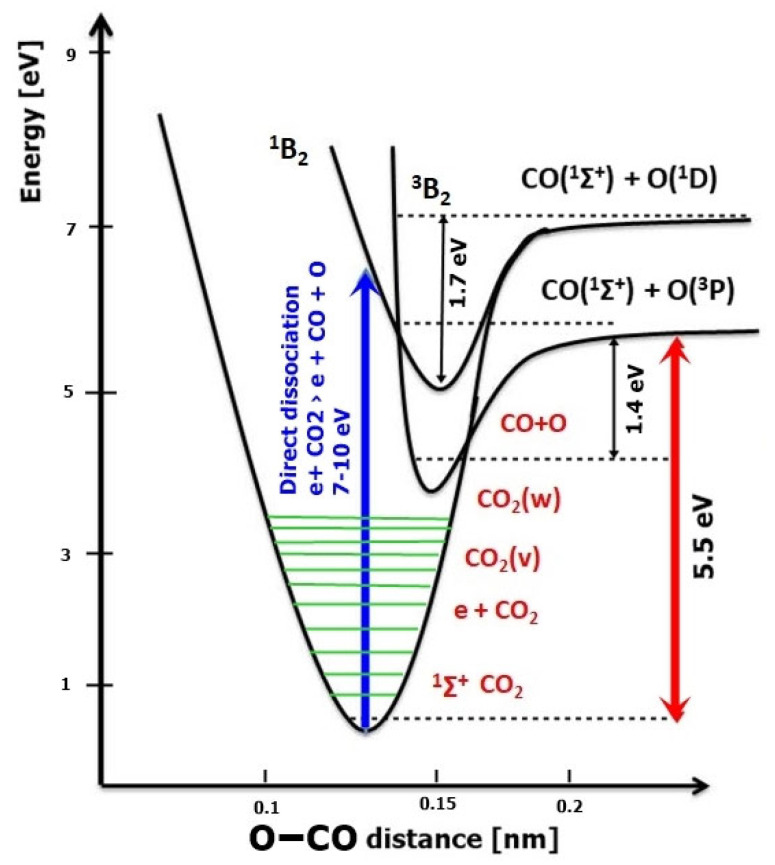
CO_2_ electronic and vibrational levels, stepwise vibrational excitation, and direct dissociation by electron impact [66].

CO_2_ can be dissociated in low-pressure plasmas through the direct impact mechanism, producing CO and O in excited electronic states requiring at least 7 eV, as shown in Figure 5:

CO_2_^*^ (^1^Σ+) → CO(^1^Σ^+^) + O(^1^D). (1)

An indirect dissociation route much more encountered in laboratory plasma discharges is the step-by-step vibrational excitation where low-energy electrons (~1 eV) transfer their energy to the asymmetric stretch vibrational mode of CO_2_ [61,62,63,64,65]:

CO_2_^*^ (^1^Σ^+^) → CO_2_^*^ (^3^B_2_) → CO(^1^Σ^+^) + O(^3^P). (2)

Through energy exchange to a highly-vibrational level, the CO_2_ molecules can reach the threshold of ~5.5 eV to produce dissociation as a second kinetic order. The non-adiabatic primary dissociation route (2) has a threshold activation energy much lower than the straightforward adiabatic dissociation route (1) (marked in Figure 5 with a blue arrow) and, consequently, can be exponentially faster. This indirect vibrational excitation mechanism is much more efficient, up to 97% in some types of low-temperature discharges [66], and, in addition, has a much lower energy threshold, $T_{e}=1$ eV [67,68]. Moreover, the vibrational excitation through steps that happens by the quantum exchange mechanisms causes a second dissociation process. Atomic oxygen in the fundamental electronic state O(^3^P) is able to participate in a secondary reaction to produce secondary CO and O [66]:

O + CO → CO + O_2_ (3)

This reaction then is faster than the recombination of atomic oxygen in three body paths (O + O + M → O_2_ + M) and allows a second CO molecule and O_2_ per event in the condition of vibrational temperature $T_{v}\geq \;0.1$ eV [68].

Another possibility is when the electron energy is much higher than the ionization potential of a CO_2_ molecule, and the splitting through dissociative ionization process can take place through several channels:

e^−^ + CO_2_→ e^−^ + e^−^ + CO^+^ + O, (4)

e^−^ + CO_2_→ e^−^ + e^−^ + C^+^ + O_2_, (5)

e^−^ + CO_2_→ e^−^ + e^−^ + O^+^ + CO, (6)

e^−^ + CO_2_→ e^−^ + e^−^ + O_2_^+^ + CO, (7)

The secondary electrons from these paths can also contribute to the vibrational dissociative mechanism with a lower energy threshold.

Negative oxygen ions can be formed in CO_2_ plasma by mechanisms of dissociative attachment and dissociative recombination as in the following reaction:

e^−^ + CO_2_→ e^−^ + e^−^ + CO + O^−^ (8)

e^−^ + CO_2_ → CO + O (9)

This reaction has a relatively reduced energy barrier (3.85) eV [65], but the cross-section for electron attachment is about $\sigma \approx 0.1–0.4\times 10^{-22}$ m^2^ [69], which is three orders of magnitude smaller than the vibrational cross-section; therefore, this mechanism cannot improve the dissociation fraction too much. However, the reaction is important for the overall plasma charge balance. The reaction products are not necessarily CO and O. It could turn out from recombination of C + O_2_, C + O + O, or simply CO_2_ again. Formation of electronically excited states of CO and O may also be possible [70,71]. The reaction (9) and its variants play a significant role only in the case of high electron temperatures (20–40 eV [70]) since it is necessary to exceed the ionization potential of CO_2_, of 13.3 eV. Among these categories, vibrational excitation has a rate of at least one order of magnitude compared to the other excitation channels [72,73]. A line of the carbon ion identified at C+ (724.13 nm) (Figure 1) could be a sign of a secondary CO dissociative ionization channel:

e^−^ + CO→ e^−^ + e^−^ + C^+^ + O (10)

with a relatively high energy threshold of 9.144 eV/mol., or a recombination path of CO_2_ molecule such as

CO^+^ + CO→ C^+^ + CO_2_ (11)

### 3.2. Estimate Rate of Dissociation

An essential question is how effective a coaxial plasma gun would be in dissociating CO_2_.

Looking at the temperature and electron density profiles measured with the triple Langmuir probe (as shown in Figures 10 and 11 of the Section 3.2), we observe that in the first stage of all discharges, the peak electron temperatures are high enough (i.e., 11–14 eV) in order to dissociate CO_2_ directly by electron impact. In the second stage of the discharge, the temperature is somehow lower in a broader range, from 3 to 9 eV, and here dissociation through electronic excitation of vibrational levels is rather prevailing. We can estimate the number of CO_2_ molecules dissociated per unit of time and volume as follows:

(12) $$dn_{\;1}/dt=K_{dir}n_{e}\;n_{g},$$

where $K_{dir}≅\sigma_{dir}\;v_{th}\;$ is the dissociation rate constant by direct electron impact; $n_{g}$ is the neutral gas density, is the dissociation cross section by direct electron impact, and $v_{th}=\sqrt{\frac{8k_{B}T_{e}}{\pi m_{e}}}$ is the mean speed of electrons. We use in our calculations $\sigma_{dir}=10^{-21}$ cm^2^ at $T_{e}=11$ eV and $\sigma_{dir}=3\times 10^{-21}$ cm^2^ at 14 eV [66]. In our first case, we get $K_{dir}^{1kV}=2.2\times 10^{-15}$ m^3^ s^−1^, which leads to ${\left.\frac{dn_{1}}{dt}\right|}_{1\;kV}=4.8\times 10^{29}$ particles m^−3^ s^−1^ at 1 kV. For the 2 kV shot, we have $K_{dir}^{2kV}=7.5\times 10^{-15}$ m^3^ s^−1^ and ${\left.\frac{dn_{1}}{dt}\right|}_{2kV}=2.4\times 10^{30}$ particles m^−3^ s^−1^. Here we do not account for the recombination processes and other losses by molecular collisions. The plasma volume ejected from the coaxial gun is estimated by using

(13) $$V_{pl}=\frac{v_{i}\tau_{i}\pi d_{coax}^{2}}{4},$$

where the length of the discharge gun is $v_{i}\tau_{i}$. The ion velocity is $v_{i}$~3.5 km/s and $\tau_{i}=250\;$ µs is the total pulse duration inferred from the high-speed camera frames of Figure 7 and from the discharging current and density profiles (shown in Figures 8 and 9); $d_{coax}=17$ mm is the diameter of the coaxial plasma gun. We obtain the total number of dissociation processes by the following:

(14) $$N_{dir}=\Delta n_{1}V_{pl}\Delta t_{1},\;$$

where $\Delta t_{1}\approx 100\;$ µs is the duration of the first plasma stage: $N_{dir}^{1\;kV}=9.7\times 10^{21}$ particles and $N_{dir}^{2\;kV}=4.7\times 10^{22}$ particles for the 1 and 2 kV shots, respectively.

In terms of mass dissociated, we have $m_{dir}^{1\;kV}=0.7$ g and $m_{dir}^{2\;kV}=3.4$ g in the two cases. The corresponding energy budget required to charge up the capacitor bank is 250 J in the first case and 1000 J in the second case.

Furthermore, if we consider a repetitive operation with frequency f=240  h^−1^, one can enhance the production rate by almost three orders of magnitude, taking into account that the time required to charge the capacitor is 15 s at a constant current of 30 mA.

We can also estimate the dissociation processes in the second stage of the discharge, which is predominant through the excitation of the vibrational levels of CO_2_. Based on a total dissociation rate from all vibration levels $K_{vib}=3.9\times 10^{-16}$ m^3^ s^−1^ [71,72,73], we can estimate the dissociation rate per unit time and volume in the two cases: ${\left.\frac{dn_{2}}{dt}\right|}_{1kV}=1.5\times 10^{28}$ particles m^−3^ s^−1^ and ${\left.\frac{dn_{2}}{dt}\right|}_{2kV}=2.5\times 10^{28}$ particles m^−3^ s^−1^, respectively, considering a discharge time $\Delta t_{2}=150$ µs. The resulting mass of dissociated CO_2_ gas is then $m_{vib}^{1kV}=3\times 10^{-2}$ g and $m_{vib}^{2kV}=5\times 10^{-2}$ g, respectively. One can see that dissociation by direct electron impact is by far more efficient in our type of pulsed discharge by two orders of magnitude.

For an application on Mars, a larger enclosure would be more beneficial, and in addition, a much more efficient voltage source to raise the discharge frequency (with one pulse per second) would also boost the yield. We did most of our experiments at about 5 Torr. Moreover, it is desirable that the enclosure in which CO_2_ dissociates has a higher pressure, thus increasing the density of electrons in the plasma and, consequently, increasing the probability of dissociation.

The discharge limit of the coaxial plasma gun in this configuration is approximately 15 Torr. Furthermore, the real quantity of oxygen production could be higher through the additional mechanisms of dissociation, such as via the direct impact of electrons with CO and CO+. The increased number of spectral lines in a 2 kV discharging regime compared to 1 kV (see Figure 4) could also be the result of the dispersion of electrons energy through inelastic scattering sufficiently to produce the indirect mechanism of dissociation through stretch vibration. The ion temperature was not measured in our experiment, but based on the observations made on a similar coaxial plasma gun, we can approximate that their temperature is similar to that of the electrons [74]. Thus, one more term can be added, the same as the secondary dissociation of atomic oxygen O(3P) from relation (2). We can also envisage an oxygen production farm on Mars with several coaxial guns set to work for dissociating CO_2_ [75].

## 4. Materials and Methods

### 4.1. Experimental Setup

**Figure 6:**
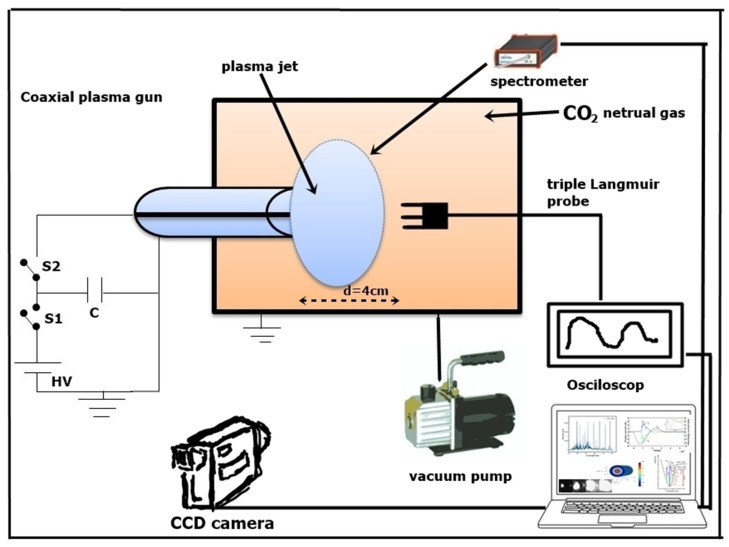
Experimental setup of the coaxial plasma gun discharging in pure CO_2_. The switches S1 and S2 control the charging/discharging state of the capacitor bank C, powered by a high-voltage (HV) source.

The scheme of the experiment is shown in Figure 6. In our setup, we were using a coaxial plasma gun to produce a plasma jet consisting of electrons and CO_2_ ions. The coaxial gun had two electrodes made of tungsten (~99.9% purity), a long centered inner rod with a diameter of 6 mm, and a coaxial outer cylindrical shell with a diameter of 17 mm. The length of the assembly was 64.5 mm. The two electrodes were mounted on a support made of polyethylene fixed on one of the flanges of the vacuum enclosure. The electrodes were extended further outside the vacuum enclosure through air vacuum seals using O-rings for electrical connections.

Before firing the plasma pulses, two vacuum pumps were used to evacuate the air from the enclosure: a preliminary fore-vacuum to reach a pressure of 10^−2^ Torr and a turbomolecular pump to obtain a high vacuum of 10^−5^ Torr. Then high-purity CO_2_ (99.998%) was introduced inside the vacuum chamber to a pressure of 1–5 Torr through a gas valve.

The coaxial plasma gun was powered by a capacitor bank with 500 µF, which was charged by a DC supply source (Glassman model EQ020R060) able to provide a steady current of 60 mA. Depending on the applied voltage (0.8–2 kV), the energy between 160 and 1000 J was stored in the capacitors. According to the Paschen law [76] for CO_2_ gas, the minimum discharging voltage of the coaxial electrode configuration was about 650 V at 5 Torr.

A pulsed current with a total duration of about 350 µs was generated in the discharging circuit while the capacitor bank was discharged. The ions were ejected in the axial direction by force J × B with velocities between 2–5 km/s, where J is the current density flowing between the electrodes, and B is the self-induced magnetic field. The discharge current was measured using a current monitor, i.e., a Rogowski coil, with a conversion factor of 0.01 V per 1 A, produced by Pearson Electronics, model 101 [77], and 2 voltage attenuators (20 and 3 dB) with attenuation factors of 10 and 1.414, respectively, produced by PICO (model TA050), all three devices being connected in series.

**Figure 7:**
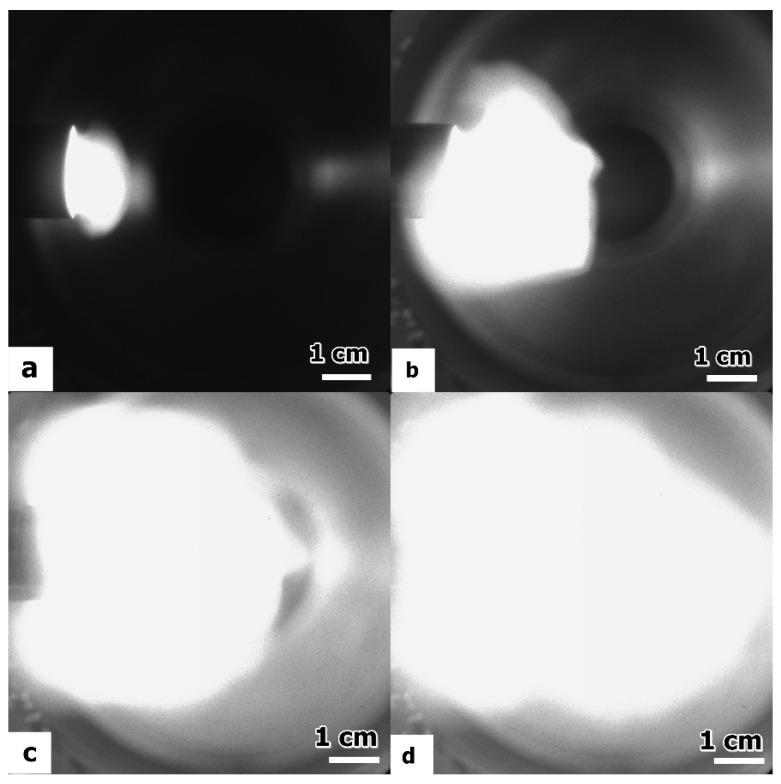
Plasma jet in CO_2_ at 5 Torr and 1 kV voltage between the electrodes, captured at different moments in time from the initiation of the discharge: (**a**) 55 µs; (**b**) 70 µs; (**c**) 80 µs; and (**d**) 90 µs.

A high-speed camera PIMAX 4 (Princeton Instruments) [78] was used to record images of the plasma jet. In Figure 7, the plasma jet expansion and evolution in time from 55 µs (in image a) to 90 µs (in image d) is shown. The camera was triggered during the ramp-up phase of the discharge current. At 90 µs, the ejected plasma is fully developed and expanded into the enclosure. Considering the jet propagation time inferred from the camera frames and the measured distance, we can deduce the jet propagation speed to be approximately 3.5 km s^−1^. The freely transversal expansion speed of the plasma flow is roughly given by the ion thermal speed. The estimate provided by this method is sufficiently accurate for plasma jets produced in plasma guns with similar electrode configurations, as shown in other works [48,79].

### 4.2. Plasma Diagnostic

The plasma density was measured with a triple Langmuir probe [80,81] positioned at a distance of 4 cm from the gun muzzle. This distance was chosen in order to keep the probe sufficiently far from the high-voltage electrode and to avoid igniting a discharge between the electrode and probe. At the same time, it is consistent with the size of the ejected plasma. Within the limit of ~4 cm, we could focus with the high-speed camera and observe that the plasma jet was fully developed.

The triple probe was made of three identical tungsten wires with a diameter of 0.6 mm and a length of 4.5 mm [81,82]. The assembly was inserted axially with the plasma jet direction. Between probes #1 and #3, a DC constant voltage from 5 to 50 V was applied, while the currents collected by these probes ($I_{1}=-I_{3}$) were measured by Pearson current monitors (model 2877) with a response of 1 V per 1 A [77]. The voltage between the floating probe #2 and the biased probe #3, $V_{diff}$, was measured with a PICO differential voltage probe (model TA041). Using the following equation [81], we can estimate the electron temperature:

(15) $$K_{B}T_{e}=\frac{V_{diff}}{ln\left(2\right)}$$

The ion saturation current is found using the following equation:

(16) $$I_{+}=I_{1}\frac{exp\left(-\frac{eV_{diff}}{k_{B}T_{e}}\right)}{1-exp\left(\frac{eV_{diff}}{k_{B}T_{e}}\right)}$$

and the electron density can be deduced from the saturation current $I_{+}$ and the electron temperature $T_{e}$:

(17) $$n_{e}=\frac{I_{+}}{exp\left(-\frac{1}{2}\right)eA_{+}\sqrt{\frac{k_{B}T_{e}}{m_{i}}}}$$

where $A_{+}$ is the ion collection area of the probe and $m_{i}$ is the mass of the CO_2_ ions.

**Figure 8:**
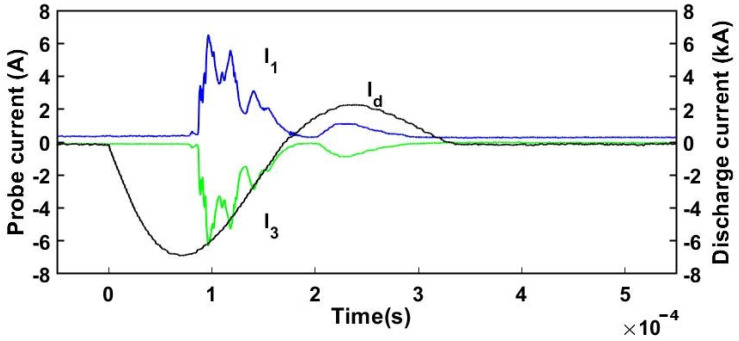
Measured plasma discharge current $I{\;}_{d}$ and triple Langmuir probe currents $I_{1}$ and $I_{3}$ at 1 kV and 5 Torr CO_2_ pressure.

**Figure 9:**
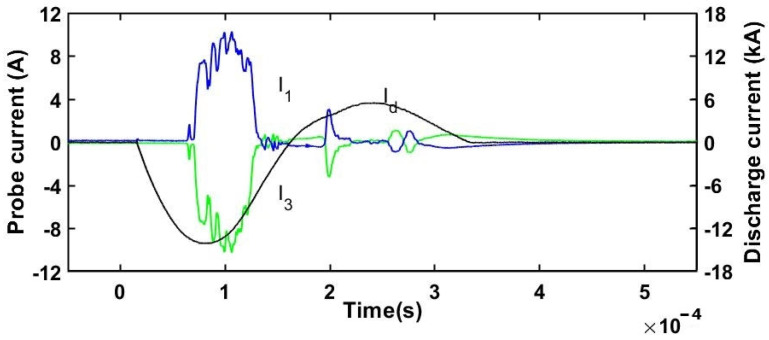
Measured plasma discharge current $I{\;}_{d}$ and triple Langmuir probe currents $I_{1}$ and $I_{3}$ at 2 kV and 5 Torr CO_2_ pressure.

**Figure 10:**
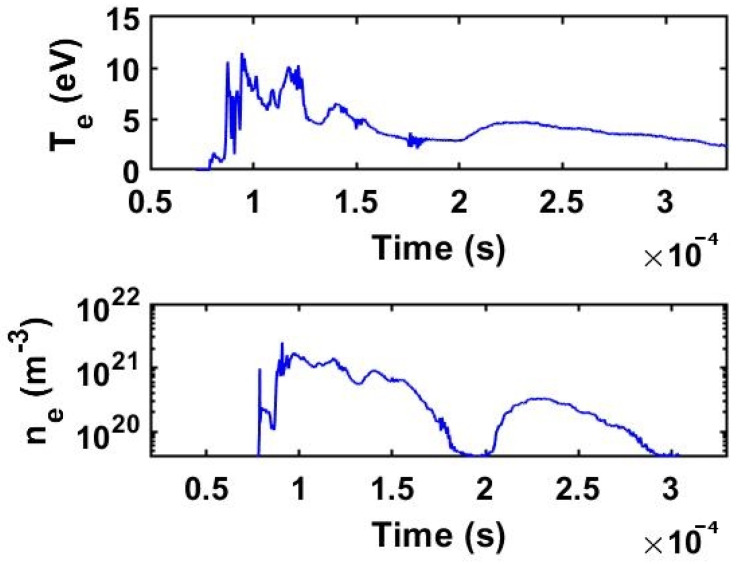
Measured electron temperature $T_{e}$ and electron density $n_{e}$ for a 1 kV shot in CO_2_ at 5 Torr.

**Figure 11:**
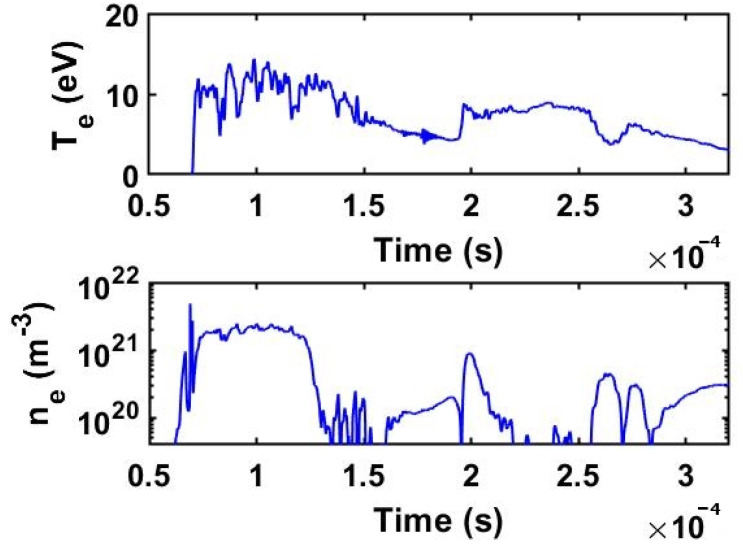
Electron temperature $T_{e}$ and electron density $n_{e}$ for a 2 kV shot in CO_2_ at 5 Torr.

The currents measured with the triple probe as well as the discharge current of the plasma gun are shown in the Figure 8 and Figure 9 and the electron density and electron temperature are presented in the Figure 10 and Figure 11.

Two representative plasma shots at 1 kV and 2 kV are presented in the following. The signals of probes #1 and #3 are delayed relative to the discharge current as they are sampling the plasma jet only when it arrives at the probe position. In the first 150 µs, the plasma discharge current peaked at approximately 7 kA at a voltage of 1 kV. The peak plasma density was in the range $n_{e}^{1kV}\;≅0.3–1.7\times 10^{21}$ particles m^−3^ for the two operating stages. At the higher voltage of 2 kV, the peak current attained 14 kA, while the electron density was $n_{e}^{2kV}\;≅0.5–2.4\times 10^{21}$ particles m^−3^. There is a clear difference between the 1 kV and 2 kV discharge shots in the sense that both electron temperature and density increase with the discharge voltage.

A note on the limitation of plasma parameter calculations is necessary. If we consider the saturation of the probe that measures the ions as given by a contribution of several ion species, e.g., CO_2_^+^, CO^+^, O_2_^+^, or other ion species resulting from early dissociation inside the ionization chamber of the plasma gun, then one could consider an average value for the ion mass $\bar{m_{i}}=(m_{CO2}+m_{O2}+m_{CO})/3$ if the proportions of these gases would be equal. In this particular case, considering that $n_{e}\sim \sqrt{m_{i}}$, according to (20), one gets mi = 5.88 instead of $\sqrt{m_{CO2+}}$ = 6.63, which is an 11.1% difference in the factor that provides the value of $n_{e}$, well within the limit of our measurements’ errors. However, we do not know from our measurements the proportions of ionic and molecular species resulting from the dissociation of CO_2_. Furthermore, the dissociated CO_2_ plasma can have a significant degree of electronegativity, which means a contribution of negative ions in the transport and the spatial distribution of charged particles, as well as on the sheath structure [83]. Details of the influence of negative ions and also quantitative measurements are left for future investigations.

In the process of plasma gun discharging, the spectral analysis of the gas composition was recorded with a spectrometer AvaSpec-ULS2048-USB2 [84] provided with a UV/VIS grating with 600 L/mm, blaze at 300 nm, and with a wavelength range from 200 to 850 nm, the spectral resolution of ~0.5 nm, and slit size 10 µm. Subsequent analyzes using the NIST database [85], the Spectrum Analyzer software [86], and the compendium book of Gaydon [87] revealed the type of atoms, ions, and molecules present in the electrical discharges.

## 5. Conclusions

We demonstrated the dissociation of pure CO_2_ at low pressure into fundamental components in intense plasma jets with peak electron temperatures of 11 to 14 eV and plasma densities of order 10^21^ particles m^−3^. The coaxial plasma gun is one of the most powerful pulsed plasma sources used in the lab with instantaneous power in the few tens of MW, with discharge currents at the 10 kA level, voltages in the 1 to 2 kV range, and pulse duration of a few hundred microseconds. Spectroscopic measurements indicated the presence of several lines of oxygen ions and ionized CO molecules. The relative intensity of some O^+^ and CO^+^ lines increased by a factor of 3 with a modest increase in the discharge voltage, from 0.8 to 1.1 kV at a pressure of 5 Torr. The number of dissociated CO_2_ molecules is $4.7\times 10^{22}$ at the highest operating voltage of 2 kV, which corresponds to a total dissociated mass of 3.4 g. We infer the total mass of atomic oxygen produced to be about 1 g for this shot. Future work will focus on quantitative measurements of the rate of produced oxygen atoms and ions and other dissociation byproducts by using a mass spectrometer.

## Data Availability

Data will be made available by request to the corresponding authors.
